# Redefining metamorphosis in spiny lobsters: molecular analysis of the phyllosoma to puerulus transition in *Sagmariasus verreauxi*

**DOI:** 10.1038/srep13537

**Published:** 2015-08-27

**Authors:** Tomer Ventura, Quinn P. Fitzgibbon, Stephen C. Battaglene, Abigail Elizur

**Affiliations:** 1 Faculty of Science, Health, Education and Engineering, GeneCology Research Centre, University of the Sunshine Coast, Sunshine Coast, Queensland, Australia; 2 Fisheries and Aquaculture, Institute for Marine and Antarctic Studies, University of Tasmania, Hobart, Tasmania, Australia

## Abstract

The molecular understanding of crustacean metamorphosis is hindered by small sized individuals and inability to accurately define molt stages. We used the spiny lobster *Sagmariasus verreauxi* where the large, transparent larvae enable accurate tracing of the transition from a leaf-shaped phyllosoma to an intermediate larval-juvenile phase (puerulus). Transcriptomic analysis of larvae at well-defined stages prior to, during, and following this transition show that the phyllosoma-puerulus metamorphic transition is accompanied by vast transcriptomic changes exceeding 25% of the transcriptome. Notably, genes previously identified as regulating metamorphosis in other crustaceans do not fluctuate during this transition but in the later, morphologically-subtle puerulus-juvenile transition, indicating that the dramatic phyllosoma-puerulus morphological shift relies on a different, yet to be identified metamorphic mechanism. We examined the change in expression of domains and gene families, with focus on several key genes. Our research implies that the separation in molecular triggering systems between the phyllosoma-puerulus and puerulus-juvenile transitions might have enabled the extension of the oceanic phase in spiny lobsters. Study of similar transitions, where metamorphosis is uncoupled from the transition into the benthic juvenile form, in other commercially important crustacean groups might show common features to point on the evolutionary advantage of this two staged regulation.

Metamorphosis is a key event in the development of many animal species where transformation from the immature to adult body form takes place. Knowledge of molecular pathways which trigger, regulate and facilitate metamorphosis remains fragmented and limited to a few model species. Crustacean metamorphosis often includes a set of dramatic post-embryonic anatomical and physiological changes which transform a disparate larva into a miniature version of the adult, usually accompanied by a change of habitat and/or behaviour^1^.

While in most decapod crustacean species, pelagic larvae metamorphose directly into benthic post larvae (PLs), a complex and gradual larval-PL transition occurs in several phylogenetic groups, including zoea-mysis transition in Dendrobranchiata, zoea-megalopa transition in Astacidea, Thalassinidea, Anomura, and Brachyura and phyllosoma-puerulus transition in palinurid and scyllarid lobsters^2^. Spiny lobsters transform from a leaf-shaped larva (phyllosoma) into an intermediary free-swimming (nektonic) phase called a puerulus (resembling the adult body plan) which transports the animal from a pelagic, oceanic stage to the benthic, juvenile development^3^. Presently, metamorphosis in spiny lobsters is considered to be the phyllosoma-puerulus transition due to vast changes in morphology^4^, although knowledge of molecular mechanisms which underlie this transition is not available.

Spiny lobsters present a unique opportunity to precisely trace the phyllosoma-puerulus transition in real time. Using the naked eye, one can sample animals on a clearly defined temporal scale in relation to transition progression. During metamorphosis in spiny lobsters, the transparent phyllosoma undergo gut atrophy where the digestive glands retract from the edges of the cephalic shield towards the center^5^ (Fig. 1). Unlike other crustaceans, this gradual retraction occurs over many hours to days, depending on species, before transition to puerulus, and due to the larval transparency it enables an accurate tracking of the exact advancement towards the transitory molt. In the Eastern rock or spiny lobster *Sagmariasus verreauxi,* this transition takes 96 hours at 20 °C^5^ (Fig. 1).

Spiny lobsters (Palinuridae) are a valuable fishery in more than 90 countries and play crucial roles in structuring coastal benthic marine communities^6^. Global landings of spiny lobsters are valued in excess of US$700M at a time when wild stocks are fully exploited and in some cases in decline due to overfishing, habitat degradation and possibly climate change^7^,^8^,^9^,^10^,^11^. Spiny lobsters are critical for sustaining rural coastal livelihoods and are an important part of the food production chain. *S. verreauxi* is one of a few closely related species which constitute the spiny lobster fishery industry in the Southern Hemisphere^12^. Recent advances in hatchery technologies have enabled closing the life cycle of this species in captivity, overcoming several hurdles including stocking densities^13^,^14^, temperature and photoperiod requirements^15^,^16^ and perhaps most challenging, the phyllosoma-puerulus transition^3^.

In spiny lobsters, this transition may occur as late as two years after hatching^17^ (Fig. 1) and is a major bottleneck for the development of a commercially sustainable aquaculture^3^,^18^. One of the challenges is the lack of knowledge concerning the biochemical triggers and molecular basis that initiates this transition^15^,^18^,^19^. From the mid-1980s global rock lobster catches were static around 80,000 t per year^20^ then declined >20% to reach their lowest level in over 30 years. Development of a commercially sustainable aquaculture practice is therefore important for securing the spiny lobster industry, to meet the demand of the ever-expanding market and mitigate the fisheries decline^21^,^22^. Recent advancement in the field has enhanced the phyllosoma-puerulus transition in *S. verreauxi* from <20% to >85% success through better understanding of the effects of abiotic factors including temperature, light and oxygen using larval respiratory technologies^15^, although cannibalism in mass culture and transition mortality from the pelagic to benthic form remains a challenge. Elucidating the molecular mechanisms underlying this transition in spiny lobsters has the potential to highlight key pathways and base a technology for synchronizing it, thereby avoiding cannibalism-induced losses.

Genome-wide transcriptional changes that occur during the shift from the pelagic to the benthic phases have been studied in only a few animals, including some *Porifera*, ascidians, gastropods and corals^23^,^24^,^25^,^26^,^27^,^28^,^29^. In arthropods, the transcriptomic changes that transpire throughout the metamorphic life stages have been examined in a limited number of hexapod species (e.g.,^30^,^31^) and more recently, in the barnacle *Balanus amphitrite*^32^, the freshwater prawn *Macrobrachium rosenbergii*^33^, the pacific white shrimp *Litopenaeus vannamei*^34^ and the Chinese mitten crab *Eriocheir sinensis*^35^. In these studies, genes with roles in mediating metamorphosis and genes related to the changes in life-style that accompany it, were found to be differentially expressed throughout metamorphosis, including newly identified transcription factors, putative hormones and G-protein coupled receptors (GPCRs). Followed by *in vivo* assays, these newly identified factors can lead to metamorphosis manipulation and novel discovery of regulatory mechanisms^36^.

Apart from the fundamental understanding that 20-hydroxy ecdysone (20-HE) is the key molt-regulating hormone and that methyl farnesoate (MF), the non-epoxidated form of the insect juvenile hormone (JH III), governs the metamorphic transition^37^,^38^, little is known regarding the molecular basis of metamorphosis in crustaceans. 20-HE activates the nuclear ecdysone receptor (ER) which hetero-dimerizes with Retinoid X Receptor (R X R) to trigger molting in arthropods^39^. Upon activation, this heterodimer enables transcription of numerous gene cascades, including other nuclear receptors^40^. Molting or ecdysis is the process through which an arthropod sheds its old exoskeleton and hardens its new one in order to grow or transform (including larval transformation and metamorphosis). Ecdysis is triggered by a sharp increase in 20-HE followed by a precipitous fall. It is generally accepted that in crustaceans MF titer oscillates for as long as the larval stages last (with larval molting mediated by 20-HE oscillation) while the metamorphic molt follows MF clearance^37^,^38^. Recently, a nuclear receptor was highlighted as a plausible juvenile hormone receptor^41^, and research is underway to elucidate the mechanism through which it regulates metamorphosis^42^. In all studied arthropods, farnesoic acid (FA) was found to be converted to MF by the abundantly expressed FA methyltransferase (FAMeT)^43^. MF is then epoxidated by CYP15A1, yielding JH III^44^, the active juvenile hormone in insects^45^. JH III role has not yet been detected in crustaceans, while MF, produced and secreted by the mandibular organ (an endocrine gland which resides at the base of the mandibles in crustaceans), is considered to be the crustacean juvenile hormone^37^,^46^.

Ventura *et al.* addressed the involvement of key regulatory genes during metamorphosis in *M. rosenbergii* using a transcriptome analysis^33^. Comparison of larval and post larval (PL) transcript levels implicated the intertwining Hedgehog and Wnt signalling pathways in crustacean metamorphosis, as well as numerous other transcripts that were found to be differentially expressed between larvae and PLs and were annotated with orthologs that had not previously been linked to metamorphosis. Based on the expression pattern, Ventura *et al.* postulated that the titer levels of the crustacean juvenile hormone MF might be dropping in PLs due to CYP15A1 up-regulation, accompanied by cessation of FAMeT expression prior to metamorphosis^33^. These results align with the central paradigm according to which the drop in MF titer level facilitates the metamorphic transition in crustaceans^37^,^38^. This study suggests that the vast phenotypic transitions which accompany metamorphosis are clearly manifested by change in gene expression and can be traced by using a transcriptome analysis.

Using a comprehensive database, which depicts the high resolution transcriptomic changes throughout the phyllosoma-puerulus transition, our research highlights key genes and pathways whose expression changes during this transition. Our findings indicate that the pathways known to regulate metamorphosis are indeed associated with the subtle puerulus-juvenile transition and not the morphologically more dramatic phyllosoma-puerulus transition. We highlight the pathways, domains and key gene families that change during the phyllosoma-puerulus transition, providing molecular support to the vast changes that occur during this transition, which is regulated by yet to be defined mechanisms.

## Results and Discussion

### Transcriptome general characteristics

From a total of more than 450 million reads generated for ten samples (duplicates of five stages throughout phyllosoma-puerulus transition in *S. verreauxi*), a total of 107,333 transcripts were assembled using Trinity. These transcripts included 69,548 contigs (mean length = 302.2, N50 = 413) and 37,785 unigenes (mean length = 506.7, N50 = 709; see length distributions in Fig. S1). An average of 70.68% (±0.85) of the reads from each library mapped to the above described transcriptome. A total of 51,620 transcripts had RPKM ≥ 1 in at least two samples. This subset of transcripts was subjected to ANOVA, performed in Partek GS, to compare the RPKMs between the five developmental stages. More than 13,000 transcripts showed fold-change of at least two between the different stages (*P* < 0.05 with FDR correction), illustrating the vast shift in transcriptional activity throughout these stages (~25.3% of the transcripts). The threshold for statistical significance was therefore set to *P* < 0.0005 with FDR correction with a fold-change of at least eight. Of the 1,169 transcripts that met these criteria (471 contigs in 338 clusters and 698 unigenes; illustrated by a heat map with hierarchical clustering, Fig. 2), 703 had Nr e-value < 10^−5^ of which 222 had hypothetical annotations and 131 predicted annotations. 466 transcripts had no Nr annotation. A total of 324 transcripts had Nt e-value < 10^−5^ of which 7 had hypothetical annotations and 64 predicted annotations. 845 transcripts had no Nt annotation. Swissprot homologs (e-value < 10^−5^), COG IDs and KO-terms were assigned to 637, 316 and 541 transcripts, respectively.

### The domains that facilitate the phyllosoma-puerulus transition

The domains which compose the predicted ORFs of the 1,169 transcripts that met the rigorous criteria of differential expression between the stages are represented as word clouds (Fig. 3). This approach has not been practised before to describe vast changes in transcriptional activity, but clearly indicates which domains are most predominant in each stage. We found that prior to the transition, in the inter-molt phyllosoma stage, a high number of domains composed the proteins that are up-regulated (Table S2). Domains with the highest occurrence were Methyl-transferase, followed by Pacifastin, followed by alcohol dehydrogenase (ADH; Fig. 3, top). These three components are primarily associated with epigenetic regulation^47^, proteinase inhibition^48^, and oxidation-reduction processing of alcohols into aldehydes and ketones^49^, respectively. Sporadic research into ADH spatial-temporal expression patterns and functions across Animalia, indicates it might have an evolutionary-conserved role in metabolising a rate-limiting step in the biogenesis of retinoic acid which controls R X R signalling pathway^50^,^51^, suggesting the R X R pathway (including molting) undergoes down-regulation prior to the phyllosoma-puerulus transition.

In the early gut retracting stage (Fig. 3, middle-left panel), the most predominant domains were filamin, chitin binding and kazal domains. Filamins are scaffold proteins which bind actin and are involved in cell motility and signalling^52^. Proteins with chitin binding domains have versatile functions, depending on the domains arrangement, but primarily they interlink the chitin layers in the cuticle, link chitin with other protein components and nucleate mineralization^53^,^54^. The chitin binding domains were found in a multitude of proteins, while the filamin and kazal domains were found to be present in tandem repeats within but a few proteins (Table S2).

In the late gut retracting stage (Fig. 3, middle-right panel) the highest domain occurrence included extensin, glucose-methanol-choline oxidoreductase (represented by the words GMC and oxred) and chitin-binding domains. Extensins have only been reported thus far in plants. Considering the marginal e-values obtained for these domains, this result should be further examined, as it might represent a new class of domains which enable cell extension and expansion through cross-linking, similar to the extensins role in plants. The GMC oxidoreductases had highly significant e-values (Table S2). GMC oxidoreductases were recently linked to defence mechanisms in beetle larvae^55^, but not to metamorphic transitions.

In the post-molt puerulus stage there were very few up-regulated domains (Fig. 3, bottom-left panel). These included the chitin binding domain and cuticle-1 domain which have so far been isolated only from calcified regions of clawed lobster and rock crab cuticles^56^. This suggests that post-molt pueruli use proteins with these domains to calcify their cuticle.

In the H-phase puerulus stage, the most predominant domains included immunoglobulins (represented by the words ig, I-set and V-set; Fig. 3, bottom-right panel). This result is biased by the fact that these domains substantially repeat within three major structural proteins of high molecular weight (Table S2). BLASTP results for these proteins gave over 99% similarity and they corresponded to Titin (partial) and Hemicentin-1. Titin functions as a molecular spring that is responsible for the muscle elasticity^57^. Hemicentin forms extracellular scaffolds that stabilize the germline syncytium, anchors mechanosensory neurons to the epidermis, and organizes hemidesmosomes in the epidermis^58^.

### Metabolism of metamorphosis regulating hormones

Intriguingly, FAMeT, which generates MF (the juvenile hormone in crustaceans^37^), was not found in the list of differentially expressed transcripts, as would be expected based on results obtained in freshwater prawns^33^. We identified a highly similar homolog in the lobster transcriptome (Unigene44448, termed here Sv-FAMeT1; Nr e-value = 2E^−168^). Using real-time qPCR we confirmed that the expression of the transcript encoding Sv-FAMeT1 is uniform throughout metamorphosis (Fig. 4A), in alignment with the results obtained in the transcriptome. We also found a highly similar isoform of the enzyme CYP15A1, which degrades MF (CL6907.Contig1, termed here Sv-CYP15A1; Nr e-value = 2.00E^−117^) and verified it is expressed primarily in the adult mandibular organ (results not shown). Using real-time qPCR we found expression of Sv-CYP15A1 is basal throughout metamorphosis and is slightly, yet significantly elevated at the puerulus H stage and more significantly elevated at the juvenile stage (Fig. 4B), in keeping with the trend observed in the RPKM values.

**Figure 1:**
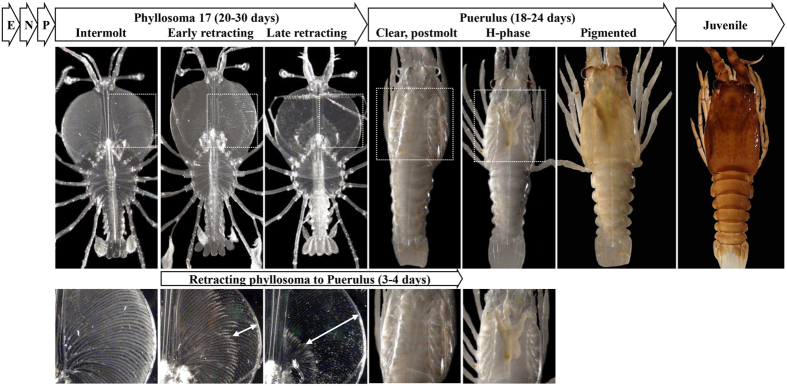
The life stages of *S. verreauxi* with emphasis on the phyllosoma-puerulus and pherulus-juvenile transitions. Following a long period of embryonic development, a short nauplius stage and 16 oceanic phyllosoma stages (E, N and P, respectively; up to 2 years, of which 6–12 months in the phyllosoma stages), the last oceanic phyllosoma stage (17) lasts for 20–30 days. Prior to molt, the gut retracts from the cephalic shield, enabling simple tracing of the molt as it occurs, with clear discernment between early and late retracting individuals. Following a complete retraction (that prolongs up to 24 hours), a molt occurs within 3 days eventuating in a clear, non-feeding, nektonic puerulus. The puerulus swims to shore, develops a functional digestive system, leading to coloration of the hepatopancreas (H-phase puerulus) and later to cuticular pigmentation (pigmented puerulus). Following another molt, a juvenile emerges. Bottom panels show higher magnification of the retraction in the phyllosoma 17 and the digestive system generation in the puerulus. Arrows indicate gut-retracted region of the cephalic shield. Photographs were taken by Dr Quinn P. Fitzgibbon and processed by Dr Tomer Ventura.

**Figure 2:**
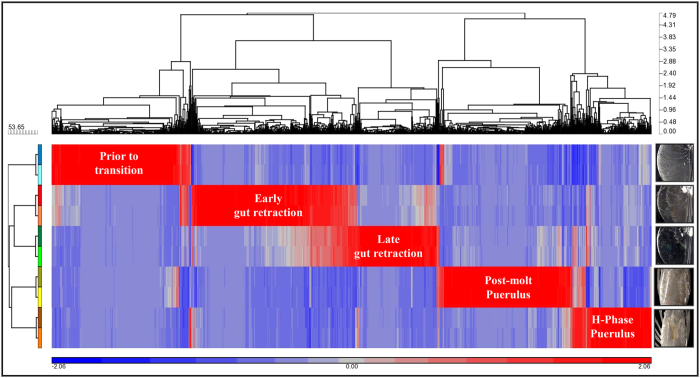
Hierarchical clustering of genes with highly significant differential expression between the five well-defined stages prior to, during and after metamorphosis in *S. verreauxi* (red = up-regulated, blue = down-regulated). A distinct pattern is observed where groups of genes are clustered together in each stage with high overlap between the two metamorphosing larval stages. The horizontal clustering is based on expression pattern across all samples. The ten samples are shown on the vertical axis, where the duplicate samples of each stage cluster tightly together. Photographs were taken by Dr Quinn P. Fitzgibbon and processed by Dr Tomer Ventura.

**Figure 3:**
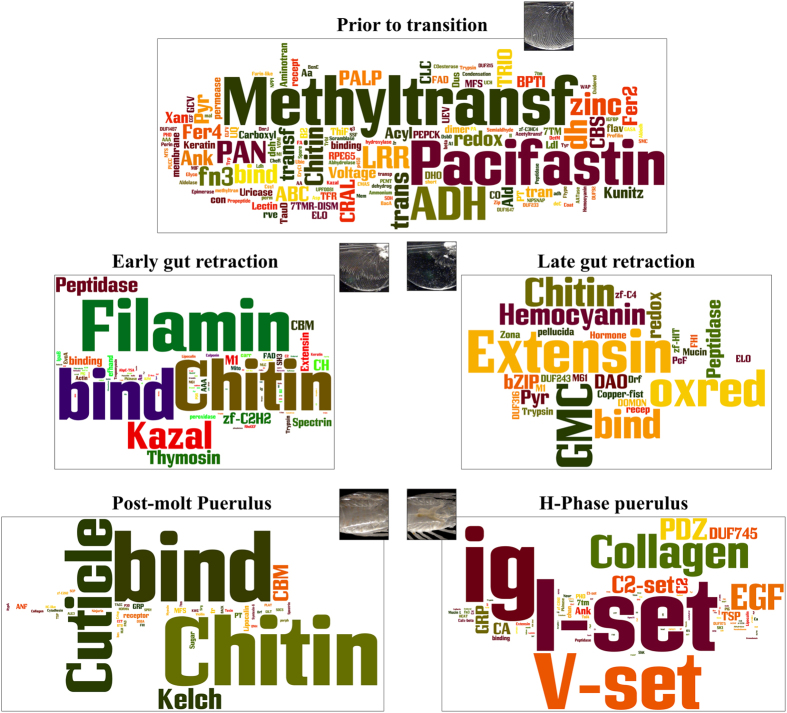
Word cloud depiction of domains prevalence during the phyllosoma to puerulus transition. Font size represents number of domains which occur in each of the five sub-stages of the transition which served for constructing the transcriptome, including intermolt phyllosoma (top), early and late gut retracting phyllosoma (middle, left and right, respectively), and pueruli post molt (bottom left) and later, when the hepatopancreas becomes visible (bottom right). Photographs were taken by Dr Quinn P. Fitzgibbon and processed by Dr Tomer Ventura.

In light of the recent finding in the giant freshwater prawn *M. rosenbergii*, where CYP15A1 was absent in larvae and present in post larvae, with the opposite trend of FAMeT^33^, these results indicate that a different mechanism regulates the transition from phyllosoma to puerulus in *S. verreauxi*, while perhaps a similar mechanism applies for the metamorphosis of the final puerulus H phase to the juvenile stage (Fig. 1). A sharp increase in CYP15A1, and/or decrease in FAMeT1 might serve as an indicator of the MF-regulated transition in other crustacean species with bi-phasic metamorphic transitions, as found in caridean shrimp zoea-decapodid and decapodid-juvenile transitions as well as in crab zoea-megalopa and megalopa-juvenile transitions^2^. A comparative analysis of the wide array of genes that we identified as differentially expressed between the phyllosoma and puerulus stage in the current study might contribute towards elucidating these hard to define transitions.

**Figure 4:**
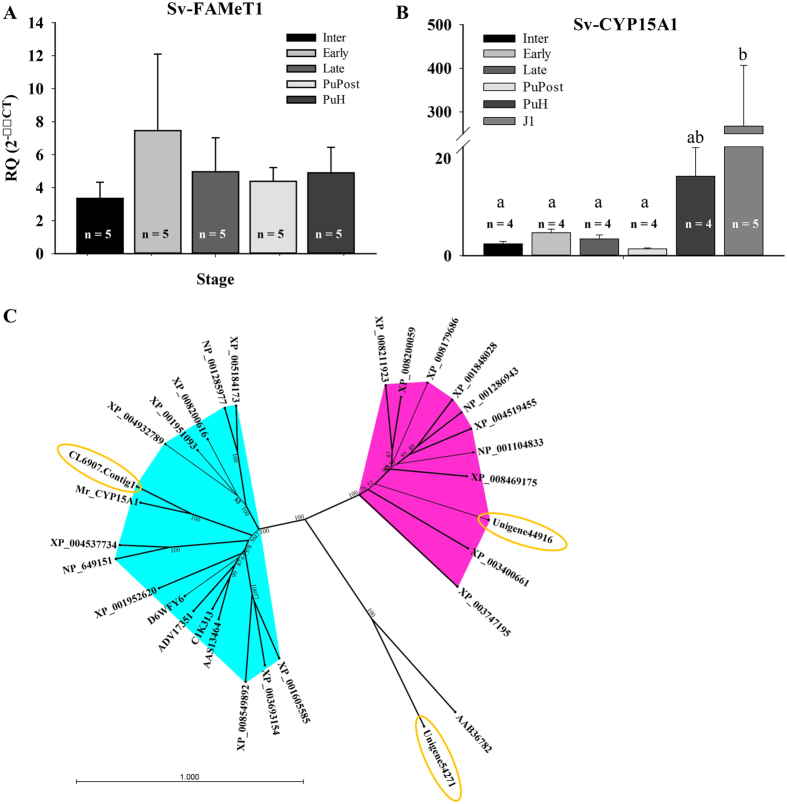
Analysis of the transcripts which encode the enzyme that metabolizes the juvenile and molt hormones. (**A**) *Sv-FAMeT1* relative quantification shows no significant difference between the metamorphic stages. (**B**) *Sv-CYP15A1* expression is basal throughout the phyllosoma-puerulus transition and is significantly elevated at the puerulus H stage (PuH; *P* < 0.05), with much higher expression in the juvenile stage (J1; *P* < 0.001). (**C**) Radial phylogram of Sv-CYP15A1 (circled, left) which branches with CYP15A1 as well as the closely-related CYP303A1 and CYP305A1 of other arthropods (left branch, highlighted in blue), to the right (highlighted in pink) is Sv-CYP307A1 (circled), branched with other arthropods CYP307A1. Sv-CYP6A2 (bottom, circled) branches with the out-group, the fruit fly CYP6A2 (please refer to Table S1 for list of proteins used in the tree). Bar represents amino acid substitutions per site.

Following the expression of genes showing the same trend of RPKMs observed for CYP15A1 (elevated in the puerulus H phase), we found a highly similar isoform of the enzyme CYP307A1 (Unigene44916, termed here Sv-CYP307A1; Nr e-value = 4.00E^−99^; expression pattern validated via qPCR in Fig. S2) that is a crucial part of the active molt hormone 20-HE biosynthetic pathway^59^,^60^. This finding further strengthens the notion that different mechanism applies for the phyllosoma-puerulus transition. In order to phylogenetically annotate the two lobster CYPs, we generated a phylogenetic tree including several CYP15A1, the closely-related CYP303A1 and CYP305A1 and also CYP307A1 identified in other arthropod species. We chose the fruit fly *Drosophila melanogaster* CYP6A2 as an out-group and included its lobster ortholog (Unigene54271, termed here Sv-CYP6A2; Nr e-value = 3.00E^−111^) in the tree. The CYP6A2 outgroups separated the other sequences into two distinct branches, one including all CYP307A1 sequences, including also the one identified in lobster, and the other included the closely-related CYP15A1, CYP 303A1 and CYP 305A1 sequences. The predicted lobster Sv-CYP15A1 branched with the freshwater prawn *M. rosenbergii* Mr-CYP15A1 (Fig. 4C).

The molecular changes during phyllosoma-puerulus and puerulus-juvenile transitions suggest that MF is not driving major body-form changes but mechanisms related to the development of internal organs, particularly the gut, to allow processing of food and benthic existence, as well as calcification of the exoskeleton. While MF is cleared at the last larval to juvenile transition, in agreement with other crustacean species, it does not change during the most dramatic morphological changes as in other crustacean species, suggesting that MF functionality may not be necessarily related to external morphological changes but to other internal mechanisms which allow juvenile existence.

From an evolutionary perspective, there are suggested advantages for highly fecund marine species such as spiny lobsters in prolonged oceanic stages. These enable stretching the habitat confinements and acquisition of new ecological niches, reducing resources competition with later life stages, breaking the parasite cycle and avoiding benthic predators^61^,^62^. On the other hand, the energetic cost of high fecundity is a prerequisite in order to compensate for the high mortality associated with this strategy^62^,^63^. The requirement of spiny lobster juvenile settlement close to shore is thus mediated by the transitional puerulus phase. Acquiring a molecular mechanism to trigger the oceanic to nektonic transition is thus a key factor in the evolution of the extraordinarily lengthy spiny lobsters development and perhaps even speciation, as the prolonged development fuels variation by exposing individuals to more variable selective forces. Shrimps and crabs also exhibit a dual transition mode with uncoupled metamorphosis and transition to the benthic juvenile form^2^. Examination of the molecular mechanisms which underlie the two stage transition in these groups, taking into consideration their life history, may explain the selective forces that drove the dual mechanism developed in spiny lobster metamorphosis regulation.

### Metamorphosis-regulating hormone receptors

R X R and ER have been shown in insects to include several isoforms with varying levels of expression throughout metamorphosis. These two components dimerize in insects to form an active nuclear receptor that activates a suit of genes that are regulated by retinoic acid and 20-HE during this process^39^. Orthologs were also identified in crustaceans and showed varying expression following autotomy^64^. A cluster of five contigs (CL4526, termed here Sv-R X R; expression pattern validated via qPCR in Fig. S2) was identified with high similarity (91% over the entire ORF) to the American lobster (*Homarus americanus*) R X R (GenBank accession number AGI15961). One of the contigs was found to have a shorter ORF with high similarity (93%) to the shorter *H. americanus* R X R splice variant (GenBank accession number AEA29832). All five contigs showed basal expression throughout the metamorphic stages with no significant differential expression (RPKMs 0.59–2.27). A cluster of four contigs (CL5835, termed here Sv-ER; expression pattern of contig 2 was validated via qPCR in Fig. S2) was found to putatively encode an ER with high similarity (89%) to *H. americanus* ER (GenBank accession number AEA29831). The ER expression pattern followed the same trend of the putative R X R. The exact identity of the receptor of juvenile hormone and MF is debated, with some recent evidence supporting the possibility of the Methoprene-tolerant receptor (MtR) being the one in insects and *Daphnia*^41^. We identified an MtR homolog (Unigene2413, termed here Sv-MtR) that shares overall low similarity with other previously identified MtRs (up to 38% identity and 56% similarity in amino acid sequence). Nevertheless, it is the first MtR to be reported in any malacostracan crustacean species, which might explain the low similarity. The domain structure of Sv-MtR is similar to that found in insects and *Daphnia*. Like Sv-R X R and Sv-ER, Sv-MtR too did not show significant fluctuation in RPKMs during the metamorphosis stages. Taken together with the finding that the enzymes which metabolize 20-HE and MF change significantly at the puerulus H phase, prior to transition to juvenile, this indicates that a different mechanism to that previously reported underlies the phyllosoma-puerulus transition in spiny lobsters.

### Structural proteins

Amongst the 1,169 differentially expressed transcripts (*P* < 0.0005; fold-change ≥ 8), seven components of the extracellular matrix-receptor interactions pathway were identified, as well as seven in the pathway of actin cytoskeleton regulation and eleven components of the focal adhesion pathway. This high coverage is in keeping with the vast structural rearrangements that accompany the phyllosoma-puerulus transition process, which is also manifested by sub-cellular cytoskeletal rearrangements that were previously postulated to modulate trafficking of ecdysteroids^65^. The highest proportion of transcripts that changed throughout metamorphosis are those that putatively encode proteins related to cuticle (96 transcripts), followed by calcification proteins (20 transcripts, of which 6 are cuticle-calcification protein homologs) and chitin binding proteins (17 strongly chitin-binding protein homologs). Interestingly, chitinases were not identified as differentially expressed, suggesting that the same chitinases are utilized in phyllosoma and pueruli, while the cuticular rearrangements that occur throughout the transition, are facilitated by other factors. Another option is that chitinase activity is regulated post-transcriptionally.

### Zona pellucida domain-containing proteins

**Figure 5:**
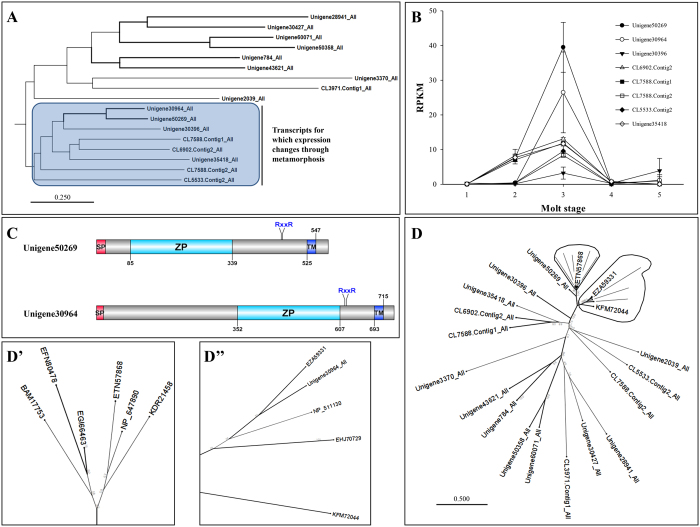
Zona pellucida domain containing proteins (ZP) characterization. (**A**) Lobster ZP domain phylogenetic tree show distinct clustering of the eight transcripts that show differential expression throughout metamorphosis (boxed) from those who don’t. (**B**) ZP transcripts with differential expression all show same trend; no expression in inter-molt phyllosoma prior to metamorphosis (stage 1), followed by a sharp incline in phyllosoma at the start of the metamorphic molt (20% gut retraction; stage 2), a decline in late stage metamorphosing phyllosoma (80% gut retraction; stage 3), with no expression in post-metamorphosis pueruli (stage 4) and H-pahse pueruli (stage 5). (**C**) The architecture of the two most prominently expressed during metamorphosis is similar to the *Drosophila* ‘dusky’, ‘dusky-like’ and ‘miniature’, all associated with wing morphogenesis and defining the apical cell shape during formation of the cuticle^67^. The putative protein starts with a short signal peptide (SP), followed by the ZP domain, a conserved RxxR motif which might enable post-translational cleavage of the protein and a transmembrane domain (TM) at the C’. (**D**) Radial phylogram of the lobster ZP domains with ZP domains of various ‘dusky’, ‘dusky-like’ and ‘cucticlin-1’ from insects localize the two most prominently expressed transcripts with the insects (see magnification in Fig. 5D’ and D”; please refer to Table S1 for list of proteins used in the tree). Bar represents amino acid substitutions per site.

Several transcripts encoding proteins containing a zona pellucida (ZP) domain were found to be highly expressed during the phyllosoma to puerulus transition. Recent findings show that ZP domain containing proteins are important for organizing the link between cell membranes and extra-cellular matrix during morphogenesis^66^. A screening of the entire list of coding regions in the transcriptome highlighted seventeen putative proteins which encompass a ZP domain. Multiple sequence alignment of the ZP domains, followed by generating a phylogram, highlighted that the differentially expressed transcripts cluster independently to those who do not (Fig. 5A). The RPKMs detected for each of these differentially expressed transcripts is given in Fig. 5B (expression patterns of isoforms 1 and 2 were validated via qPCR in Fig. S2). Most transcripts show higher RPKMs during gut retraction with elevated levels at late gut retraction (Fig. 5B). The domain architecture of the two transcripts that are most highly expressed, primarily during metamorphosis (Fig. 5C), resembles the architecture of the Drosophila ‘Dusky’ and ‘Miniature’ proteins which were shown to relate to wing morphogenesis and defining the apical cell shape in the first cuticular layer in the adult fly^67^. Remarkably, when aligning the ZP domains identified in lobster with several of the ZP domains identified in various insects, the ZP domains of these two prominently expressed transcripts cluster with the insect ‘dusky’ and ‘miniature’ (Fig. 5D). These results suggest there is an evolutionary conserved structure-function relationship between the ZP domain, the overall architecture of the ZP domain containing protein, and morphogenesis. In the present study a clear distinction between two clades of ZP domain containing proteins was identified, one which is related to morphogenesis and another that might function in different mechanisms. These results coincide with the notion that ZP domains are part of other proteins that function in other processes, such as oocyte maturation^68^.

### Morphogenesis coordination or immunity?

**Figure 6:**
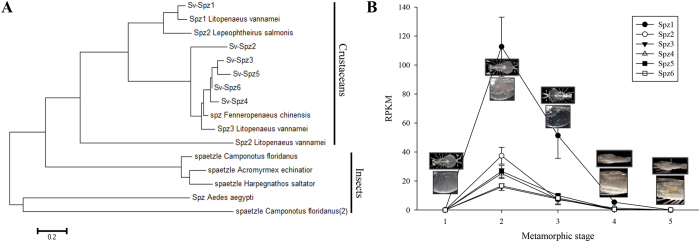
Spatzle phylogenetic analysis and expression throughout metamorphosis. (**A**) Spatzle phylogeny: Spatzle genes identified in the Eastern rock lobster all branch with other spatzle genes identified in crustacean species, distinct from those identified in insects (please refer to Table S1 for list of proteins used in the tree). Bar represents amino acid substitutions per site. (**B**) Spatzle genes all follow the same expression pattern throughout metamorphosis (left to right): no expression in inter-molt phyllosoma prior to metamorphosis (stage 1), followed by a sharp incline in phyllosoma at the start of the transition molt (20% gut retraction; stage 2), a decline in late stage transition phyllosoma (80% gut retraction; stage 3), with no expression in post-metamorphosis pueruli (stage 4) and H-pahse pueruli (stage 5). Photographs were taken by Dr Quinn P. Fitzgibbon and processed by Dr Tomer Ventura.

A homolog of UNC93 (CL5857.Contig2; Swissprot e-value = 2.00E^−129^) was significantly enriched during gut retraction (RPKMs 60.3–76.6 in both 20% and 80% retracting phyllosoma) compared with inter-molt phyllosoma and post-molt and H-phase pueruli (RPKMs 0.26–4.1; expression pattern validated via qPCR in Fig. S2). In the worm, *Caenorhabditis elegans,* UNC93 is involved in the coordination of muscle contraction^69^, a function that is consistent with a process that requires highly coordinated muscle contractions^70^. In human, an UNC93 homolog is involved in trafficking toll-like receptors (TLRs) within the cell and is thus an important factor in the innate immune system, as this is the main role assigned to TLRs^71^. In *Drosophila*, where the TLRs were identified for the first time, apart from a role in the innate immunity against fungal and bacterial infections in adult flies^72^, TLRs were found to play a critical role in defining the dorso-ventral axis during embryogenesis^73^, showing the dual role of the TLR pathway at different life stages in *Drosophila*. The establishment of the dorsal–ventral (D–V) axis in the early *Drosophila* embryos depends on the localised secretion of Spatzle (SPZ; encoded by the spatzle (spz) gene) that forms a gradient in the developing embryo. SPZ binds the transmembrane TLRs that are uniformly distributed on the cell membrane. Active SPZ gradient (with its highest level at the ventral side) initiates a stronger signal transduction at the ventral side, which leads to activation of genes necessary for establishing the ventral fates^74^. Six transcripts encoding spatzle (SPZ) orthologs were identified in this study, termed *Sagmariasus verreauxi* spz (Sv-spz 1-6; expression pattern of *Spz1* validated via qPCR in Fig. S2). Phylogenetic analysis showed all Sv-spzs are branched with other spzs identified in crustaceans, and clearly distinct from those identified in insects (Fig. 6A). All Sv-spzs showed the same pattern of expression; absent in all tissues in the adult and juvenile individuals with differential expression throughout metamorphosis: all Sv-spzs showed no expression at the inter-molt phyllosoma, followed by high expression at the early (20%) gut retracting individuals, with decline in expression at the later 80% gut retracting individuals and reverting to no expression at the early post-molt Puerulus stage (Fig. 6B). Based on the above, we speculate that SPZ coordinates morphogenesis during rock lobsters metamorphosis. Interestingly, it was noted in *Drosophila* that genes that activate SPZ and facilitate its binding to TLRs show significant transcription during metamorphosis, in the absence of infection^75^.

### Neuroendocrine regulation and sensing

**Figure 7:**
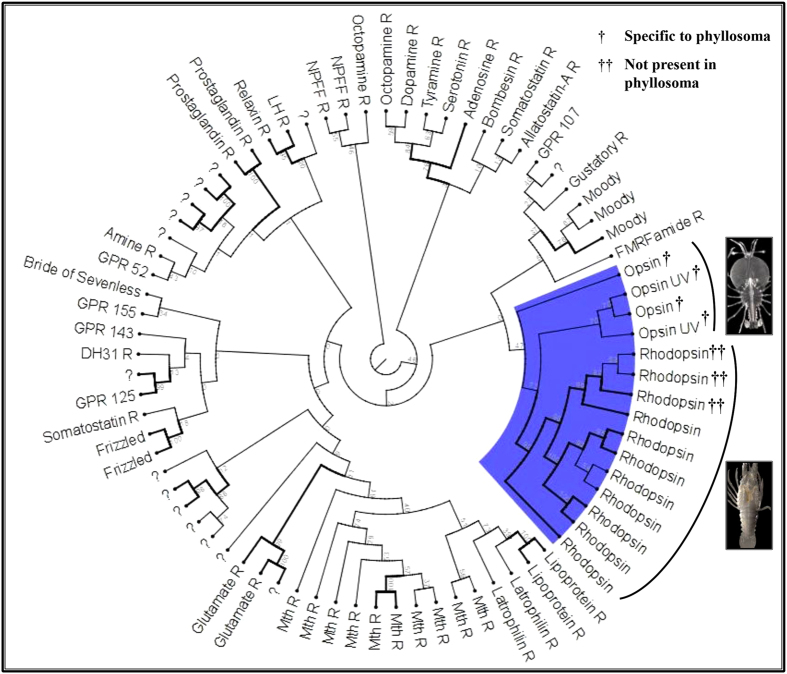
A radial phylogram of the 7TM domains of all GPCRs identified in *S. verreauxi*. Names were assigned based on comparison to highly similar sequences characterized primarily in insects and *Daphnia*, except where ambiguous (question marks). The branch of photoreceptors is shaded. All four GPCRs related to daylight vision are specific to phyllosoma. These cluster with 11 rhodopsins, 3 of which are specifically present at later stages (absent in phyllosoma), reflecting the diurnal to nocturnal shift in the phyllosoma to puerulus transition. Photographs were taken by Dr Quinn P. Fitzgibbon and processed by Dr Tomer Ventura.

G protein-coupled receptors (GPCRs) convey the signal of extracellular ligands (including photons, odours, pheromones, hormones and neurotransmitters) intra-cellularly^76^. Of the 76 GPCRs we identified and partially annotated in *S. verreauxi* (Fig. 7), 11 were annotated as rhodopsins and another 4 as opsins. Seven of which (Nr e-values 3.00E^−130^–5.00E^−180^) were differentially expressed throughout metamorphosis, including three rhodopsins (involved in night vision) up-regulated in the puerulus H phase. Interestingly, these rhodopsins were found also in the eyestalk of later juvenile stages, indicating the shift in activity to a nocturnal behaviour pattern is accompanied by expression of another set of GPCRs. The four opsins were specific to phyllosoma and included two homologs of an ultraviolet-sensitive opsin from the honey bee *Apis mellifera* (Swissprot e-value = 1.00E^−124^), suggesting the larval eye can detect very short wave-lengths. These results are in keeping with the observed transition from apposition (daylight adapted) to superposition (night vision adapted) eye formation between larval and adult stages of the closely related Southern spiny lobster *Jasus edwardsii*^77^. Moreover, our data further exemplifies how fundamental changes such as the eye structure and function occur at the phyllosoma-puerulus transition, uncoupled from the later, puerulus-juvenile transition.

Information regarding changes in eye-stalk peptides during crustacean larval molt is scarce^78^. From the neuropeptides recently characterized in *S. verreauxi*^79^, one crustacean hyperglycemic hormone (CHH, Unigene30324; expression pattern validated via qPCR in Fig. S2) was significantly up-regulated during metamorphosis from phyllosoma to puerulus. However, none of the Molt-inhibiting hormones showed such a trend. Further research is needed to investigate the relation of CHH and MIH to the larval molt cycle as well as metamorphosis, as the lack of studies in this field is in contrast with the vast research into the involvement of CHH superfamily of peptides in the adult molt.

## Conclusions

This research provides evidence to suggest that the levels of 20-HE and MF do not fluctuate (based on expression of their metabolising enzymes) until the H-phase puerulus, prior to transition into the first juvenile stage. The vast morphological changes accompanying the phyllosoma-puerulus metamorphic transition appear to be controlled by different and as yet undefined pathways. The definition of metamorphosis in spiny lobsters should thus be revisited and requires further research to broaden the molecular understanding of the phyllosoma-puerulus as well as puerulus- juvenile transitions. Examining gradual metamorphic transitions in other crustacean groups will enhance our understanding of the selective forces that drove this dual mechanism developed in spiny lobsters.

## Materials and Methods

### Animals

*Sagmariasus verreauxi* individuals at different metamorphic stages were cultured at Institute for Marine and Antarctic Studies under previously described parameters^13^. Prior to dissections, animals were anesthetized in ice-cold salt water for at least 10 min.

### Sample Preparation and Sequencing

Total RNA from two *S. verreauxi* individuals of each metamorphic stage selected were isolated separately with the Trizol^®^ Reagent (Invitrogen), according to the manufacturer’s instructions. Selection of individuals included five life stages: 1) inter-molt, 2) early-metamorphic and 3) late-metamorphic phyllosoma 17 (defined based on digestive glands reaching the cephalic shield edges, retracting 20% and 80%, respectively), 4) clear post-molt puerulus and 5) following another molt, where the hepatopancreas is apparent, named H-puerulus. Samples were shipped to BGI (HongKong Co. Ltd) for next generation sequencing as per manufacturer’s protocol (Illumina, San Diego, CA). Briefly, poly (A) mRNA was isolated using oligo (dT) beads and the addition of fragmentation buffer for shearing mRNA into short fragments (200–700 nt). This was followed by cDNA synthesis using random hexamer-primers in order to prevent priming bias. The short cDNA fragments were further purified using QiaQuick PCR extraction kit and resolved with EB buffer for ligation with Illumina Paired-end adapters. This was followed by size selection (~200 bp), PCR amplification and Illumina sequencing using an Illumina Genome Analyzer (HighSeq 2000, Illumina, San Diego, CA), performing 90 bp–paired end sequencing. The sequence reads were stored as FASTQ files. Overall, at least 4 Gb of cleaned data (at least 45 million reads) was generated for each of the ten samples sequenced.

### Bioinformatic analyses

Cleaning of low quality reads, assembly and annotation were done by BGI, using unpublished algorithms (BGI, HongKong Co. Ltd), Trinity^80^ and Blast2GO^81^, respectively. With the raw read FASTQ files, a transcriptome annotated via five different databases (Nr, Nt, SwissProt, COG and KEGG) was supplied by BGI. We validated the reads obtained by BGI using FASTQ/A Trimmer (http://hannonlab.cshl.edu/fastx_toolkit/index.html), which gave an output of over 99.99% of the reads untrimmed. The list of putative neuropeptides identified in the eyestalk and brain transcriptome, as previously published^79^, was retrieved via manual search in our database. Mapping was computed using CLC Genomics Workbench (CLC Bio, version 7.0.3) default parameters with the exception of similarity fraction elevated to 0.9. BAM files were then uploaded onto Partek Genomics Suite (Partek GS) where quantification was calculated, expressed as reads that map per kilobase of the transcript to which the reads map, per million reads in the total library size (RPKMs). The quantified data was adjusted to include only transcripts with RPKM ≥ 1 in at least two samples (lowering the number of transcripts from 107,333 to 51,620) and minimum RPKM value was normalized to 0.05 in order to highlight transcripts that were absent in one or more sample sets. Adjusted RPKM values were analyzed using ANOVA, performed in Partek GS, to compare the RPKMs between metamorphic stages. The threshold for statistical significance was set to *P* < 0.0005 (with FDR correction) and fold-change of at least eight. Multiple sequence alignment following by phylogenetic trees were performed using Genomics Workbench using default parameters with the exceptions of using ‘very accurate’ alignment method and bootstrap elevated to 1,000. Nodes with bootstrap higher than 75% is shown in bold.

### Word cloud

The predicted ORFs of the 1,169 transcripts, which were found to be highly differentially expressed between the sequenced stages, were identified using default parameters in the online tool ORF-Predictor (http://proteomics.ysu.edu/tools/OrfPredictor.html). The ORFs were then imported into CLC Genomics Workbench for PFAM domain search, using the PFAM domain plugin. The output of list of domains (given in Table S2) from the sequence subsets that represented up-regulation in one stage at a time was inserted into the online tool wordle (http://www.wordle.net/create), which generated word clouds that represent word prevalence by font size.

### Real-time qPCR

Real-time qPCR was performed as previously described^82^ with slight modifications. Briefly, first-strand cDNA was synthesized using Tetro cDNA Kit (Bioline) with 1 μg total RNA. The cDNA served as a template for the real-time qPCR. Relative quantification (RQ) was obtained using forward and reverse primers designed using the ‘Assay Design Center’ available at the Roche website. Primers were mixed with the cDNA and FastStart Universal Probe Master (Rox; Roche Diagnostics GmbH) and Universal ProbeLibrary Probe (Roche). A full list of primers and probes used for each gene can be found in Figure S2. Sv-18S (GenBank accession no. KF828103) served to normalize the quantification. PCR included 10 min incubation at 94 °C, followed by 40 cycles of 94 °C for 10 sec and 60 °C for 30 sec, with green fluorescence measurement on each cycle at 60 °C. Reactions were performed in Rotor-Gene Q (Qiagen). Relative quantification was calculated by equilibrating to the level of Sv-18S per sample and the sample with the lowest value (2^−∆∆CT^). Statistical analysis of the resulting RQs was performed in Partek GS using ANOVA, followed by Mann-Whitney U-test with *P* < 0.05 considered as statistically significant.

## Additional Information

**How to cite this article**: Ventura, T. *et al.* Redefining metamorphosis in spiny lobsters: molecular analysis of the phyllosoma to puerulus transition in *Sagmariasus verreauxi.* *Sci. Rep.* **5**, 13537; doi: 10.1038/srep13537 (2015).

## Supplementary Material

Supplementary Information

Supplementary dataset (Table S2)
