# Supplementary material for: Redefining metamorphosis in spiny lobsters: molecular analysis of the phyllosoma to puerulus transition in Sagmariasus verreauxi
Source: Sci Rep. 2015 Aug 27;5:13537. doi: 10.1038/srep13537 (PMC4550925; doi:10.1038/srep13537)
Supplement: Supplementary Information [file srep13537-s1.pdf]

Supplementary data for the manuscript titled: 'Redefining metamorphosis in spiny lobsters: molecular analysis of the phyllosoma to puerulus transition in *Sagmariasus verreauxi*' (Authors: Tomer Ventura, Quinn P. Fitzgibbon, Stephen C. Battaglene and Abigail Elizur)

Includes: Table S1, Figure S1-S2. Table S2 (a large dataset) is submitted separately as an excel file.

Table S1: List of proteins used in the phylogenetic trees in Figures 4, 5, 6

Species	Common name	Protein	Accession Number (NCBI GenBank)	Figure
Drosophila melanogaster	Common fruit fly	CYP6A2	AAB36782	4C
Bombyx mori	The silk moth	CYP303a1-like	XP_004932789	4C
Acyrthosiphon pisum	Pea aphid	CYP303a1	XP_001951093	4C
Tribolium castaneum	Flour beetle	CYP303a1	XP_008200616	4C
Drosophila melanogaster	Common fruit fly	Cyp303a1, isoform B	NP_001285977	4C
Musca domestica	Common house fly	CYP303a1-like	XP_005184173	4C
Macrobrachium rosenbergii	Giant freshwater prawn	Mr-CYP15A1	na	4C
Drosophila melanogaster	Common fruit fly	Cyp305a1	NP_649151	4C
Ceratitis capitata	Mediterranian fruit fly	CYP305a1-like	XP_004537734	4C
Diploptera punctata	Cockroach	CYP15A1	AAS13464	4C
Reticulitermes flavipes	Eastern subterranean termite	CYP15A1	C1K313	4C
Schistocerca gregaria	Desert locust	Methyl farnesoate epoxidase	ADV17351	4C
Tribolium castaneum	Flour beetle	CYP15A1	D6WFY6	4C
Acyrthosiphon pisum	Pea aphid	CYP305a1	XP_001952620	4C
Apis florea	Dwarf honey bee	CYP305a1	XP_003693154	4C
Nasonia vitripennis	Parasitoid wasp	CYP303a1	XP_001605585	4C
Microplitis demolitor	Parasitoid wasp	CYP305a1	XP_008549892	4C
Metaseiulus occidentalis	Predatory mite	CYP307a1-like	XP_003747195	4C
Bombus terrestris	Buff-tailed bumblebee	CYP307a1-like	XP_003400661	4C
Nasonia vitripennis	Parasitoid wasp	CYP307a1	XP_008211923	4C
Tribolium castaneum	Flour beetle	CYP307a1	XP_008200059	4C
Acyrthosiphon pisum	Pea aphid	CYP307a1	XP_008179686	4C

Figure S1: Length distribution of contigs and unigenes across all ten samples:

Key to library names:

1. Intermolt phyllosoma 17: 17D, 17D.
2. 20% retracting phyllosoma 17: 17preB, 17preD.
3. 80% retracting phyllosoma 17: 17preG, 17preI.
4. Post-molt puerulus: PuPost6, PuPost7.
5. H-phase puerulus: PuH1, PuH2.

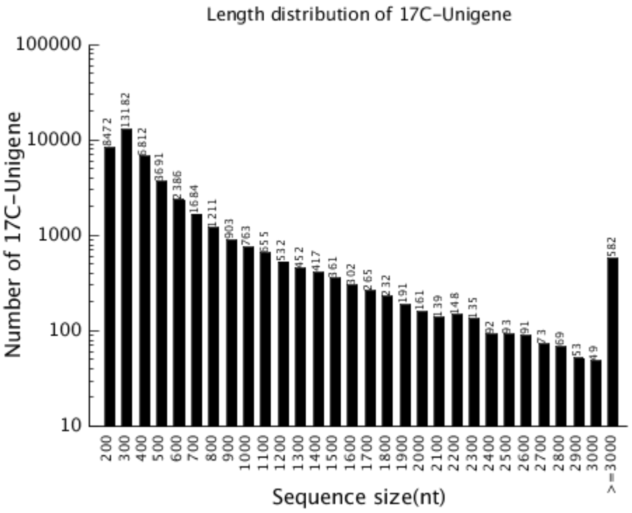

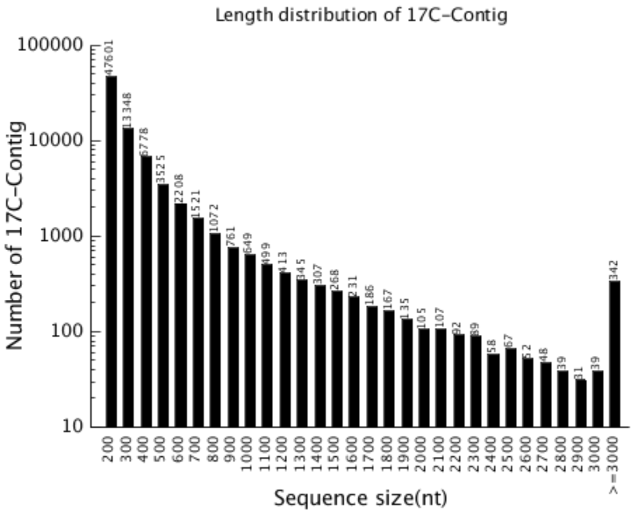

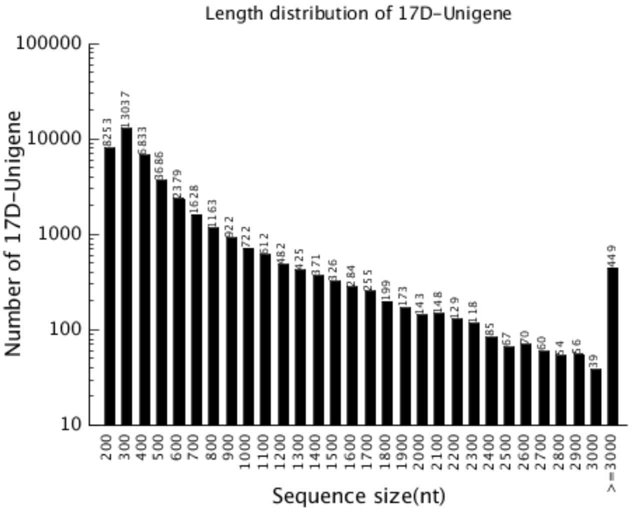

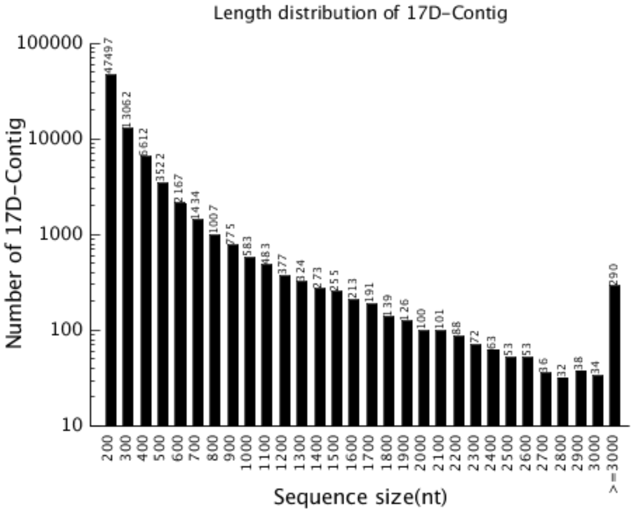

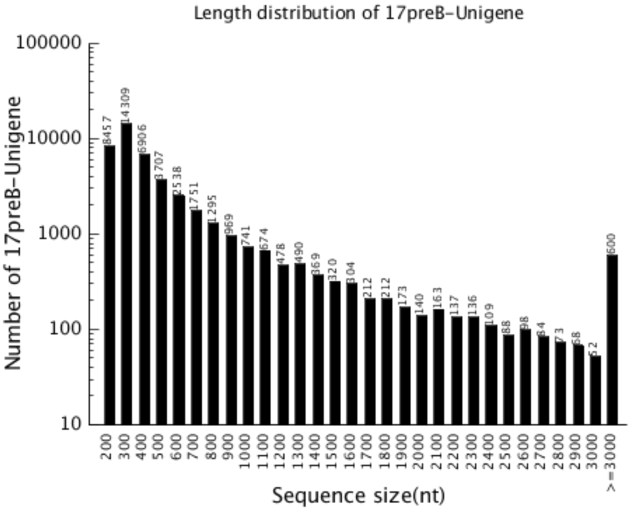

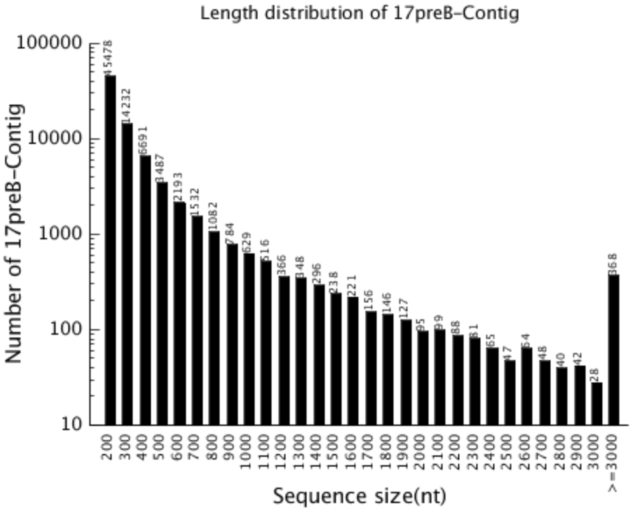

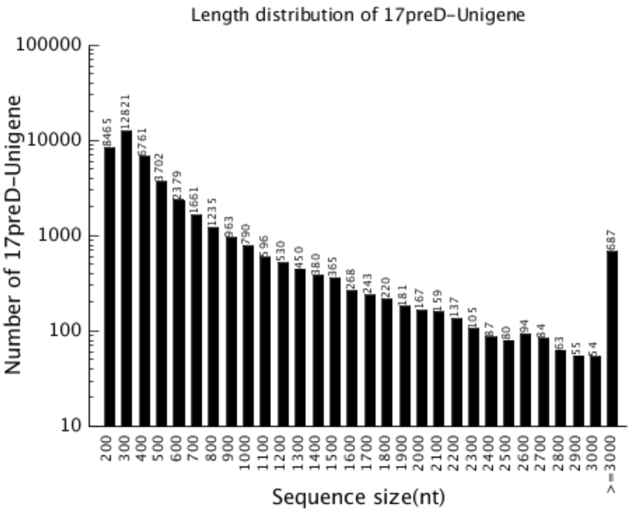
Length distribution of 17preD-Unigene
Number of 17preD-Unigene
Sequence size(nt)
100000
10000
1000
100
10
200 300 400 500 600 700 800 900 1000 1100 1200 1300 1400 1500 1600 1700 1800 1900 2000 2100 2200 2300 2400 2500 2600 2700 2800 2900 3000 >=3000
8465 12821 6761 3702 2379 1661 1235 963 790 596 530 450 380 365 268 243 220 181 167 159 137 105 87 80 94 84 63 55 54 687

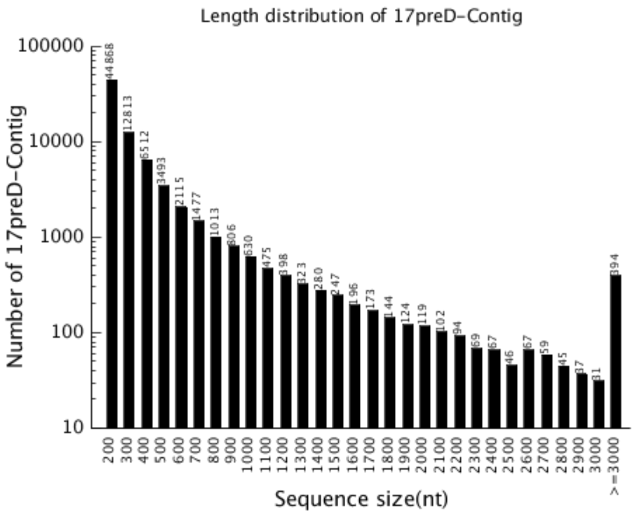
Length distribution of 17preD-Contig
Number of 17preD-Contig
Sequence size(nt)
100000
10000
1000
100
10
200 300 400 500 600 700 800 900 1000 1100 1200 1300 1400 1500 1600 1700 1800 1900 2000 2100 2200 2300 2400 2500 2600 2700 2800 2900 3000 >=3000
44868 12813 6512 3493 2115 1477 1013 806 630 475 398 323 280 247 196 173 144 124 119 102 94 69 67 46 67 59 45 37 31 394

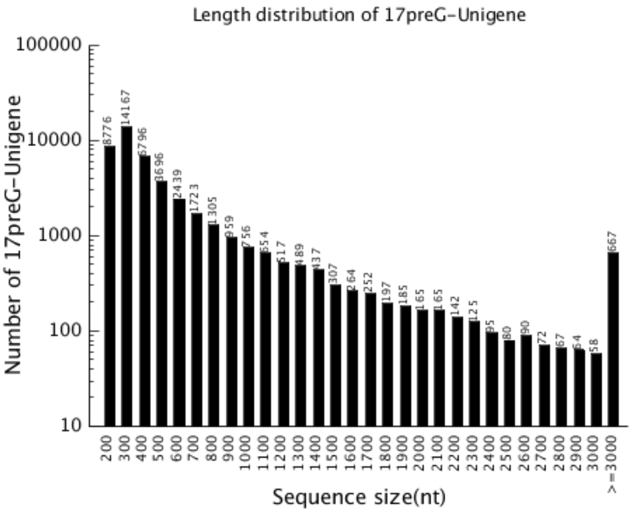
Length distribution of 17preG-Unigene
Number of 17preG-Unigene
Sequence size(nt)
100000
10000
1000
100
10
200 300 400 500 600 700 800 900 1000 1100 1200 1300 1400 1500 1600 1700 1800 1900 2000 2100 2200 2300 2400 2500 2600 2700 2800 2900 3000 >=3000
8776 14167 6796 3696 2439 1723 1305 959 756 654 517 489 437 307 264 252 197 185 165 165 142 125 95 80 90 72 67 64 58 667

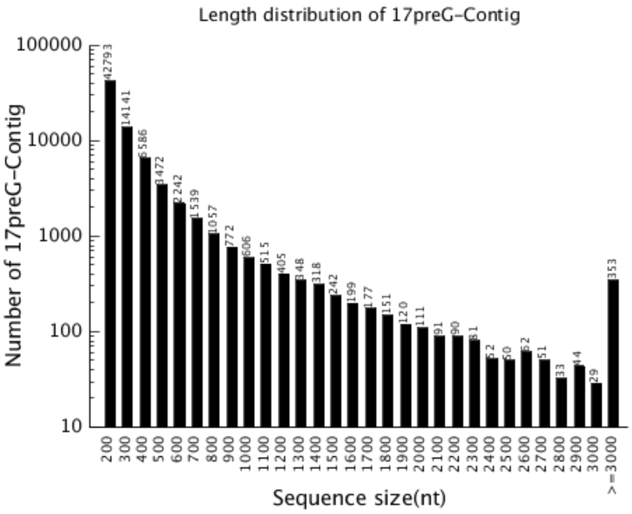
Length distribution of 17preG-Contig
Number of 17preG-Contig
Sequence size(nt)
100000
10000
1000
100
10
200 300 400 500 600 700 800 900 1000 1100 1200 1300 1400 1500 1600 1700 1800 1900 2000 2100 2200 2300 2400 2500 2600 2700 2800 2900 3000 >=3000
42793 14141 6586 3472 2242 1539 1057 772 606 515 405 348 318 242 199 177 151 120 111 91 90 81 52 50 62 51 33 44 29 353

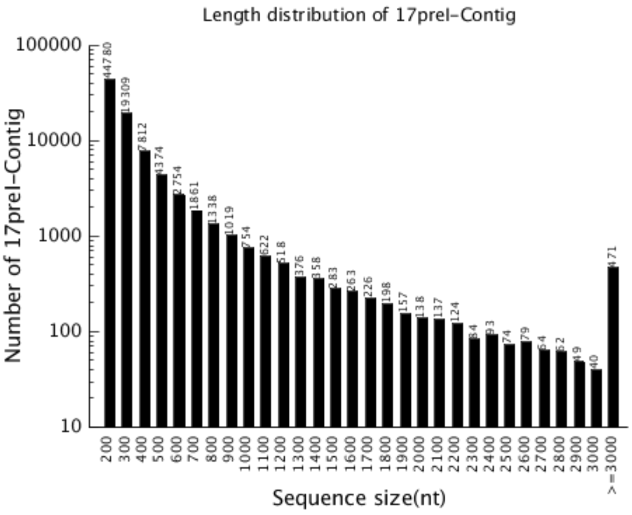
Length distribution of 17preI-Contig
Number of 17preI-Contig
Sequence size(nt)
100000
10000
1000
100
10
200 300 400 500 600 700 800 900 1000 1100 1200 1300 1400 1500 1600 1700 1800 1900 2000 2100 2200 2300 2400 2500 2600 2700 2800 2900 3000 >=3000
44780 19309 7812 4374 2754 1861 1338 1019 754 622 518 376 358 283 263 226 198 157 138 137 124 84 93 74 79 64 62 49 40 471

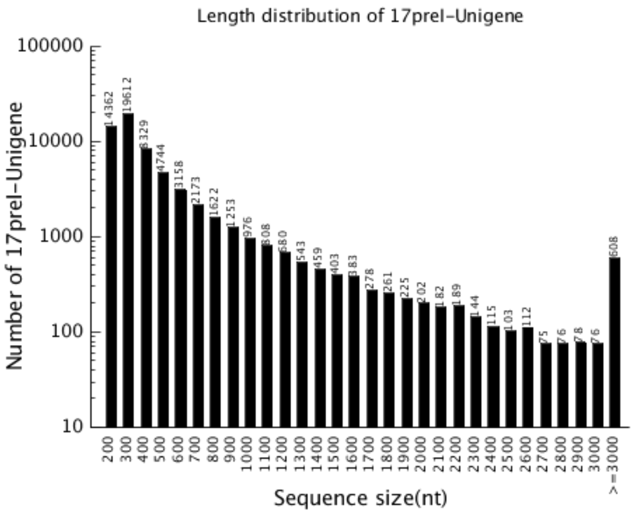
Length distribution of 17preI-Unigene
Number of 17preI-Unigene
Sequence size(nt)
100000
10000
1000
100
10
200 300 400 500 600 700 800 900 1000 1100 1200 1300 1400 1500 1600 1700 1800 1900 2000 2100 2200 2300 2400 2500 2600 2700 2800 2900 3000 >=3000
14362 19612 8329 4744 3158 2173 1622 1253 976 808 680 543 459 403 383 278 261 225 202 182 189 144 115 103 112 75 76 78 76 608

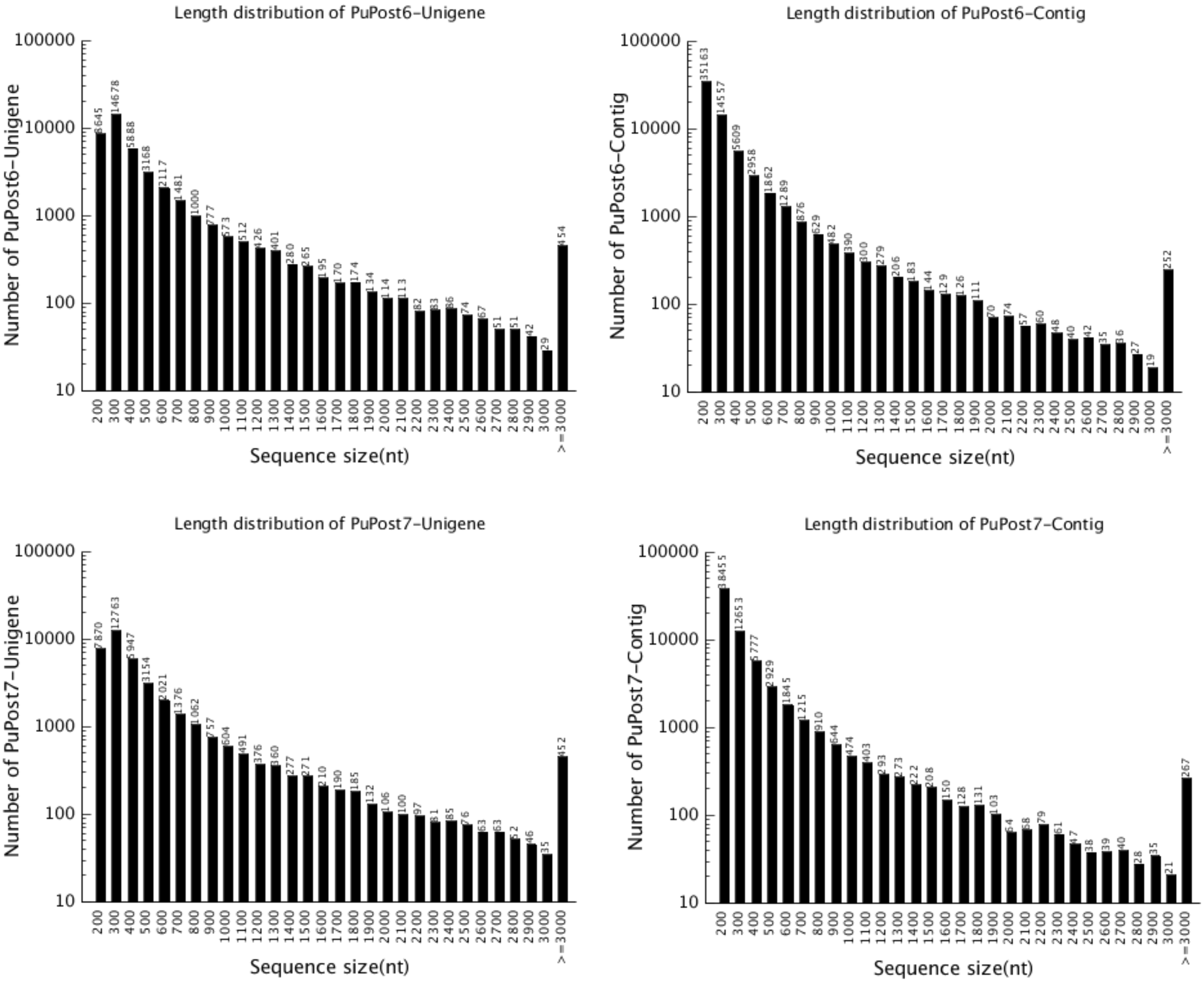
Length distribution of PuPost6-Unigene
Number of PuPost6-Unigene
Sequence size(nt)
Length distribution of PuPost6-Contig
Number of PuPost6-Contig
Sequence size(nt)
Length distribution of PuPost7-Unigene
Number of PuPost7-Unigene
Sequence size(nt)
Length distribution of PuPost7-Contig
Number of PuPost7-Contig
Sequence size(nt)

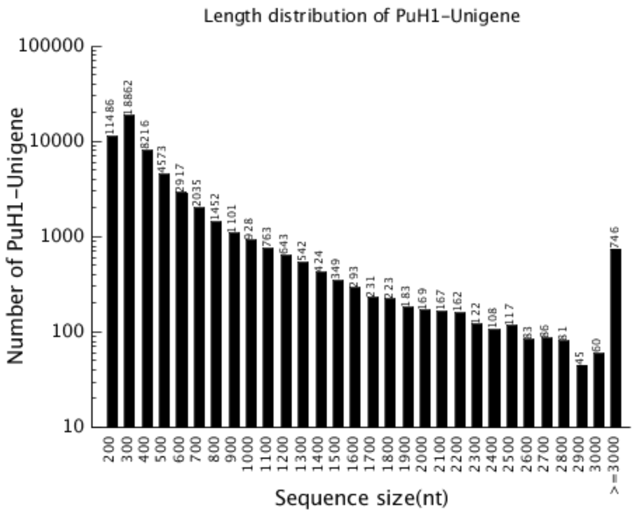
Length distribution of PuH1-Unigene
Number of PuH1-Unigene
Sequence size(nt)
11486 18862 8216 4573 2917 2035 1452 1101 928 763 643 542 424 349 293 231 223 183 169 167 162 122 108 117 83 86 81 45 60 746

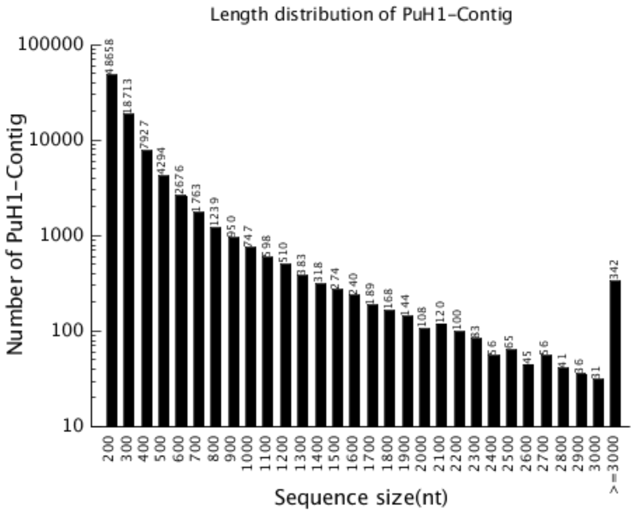
Length distribution of PuH1-Contig
Number of PuH1-Contig
Sequence size(nt)
48658 18713 7927 4294 2676 1763 1239 950 747 598 510 383 318 274 240 189 168 144 108 120 100 83 56 65 45 56 41 36 31 342

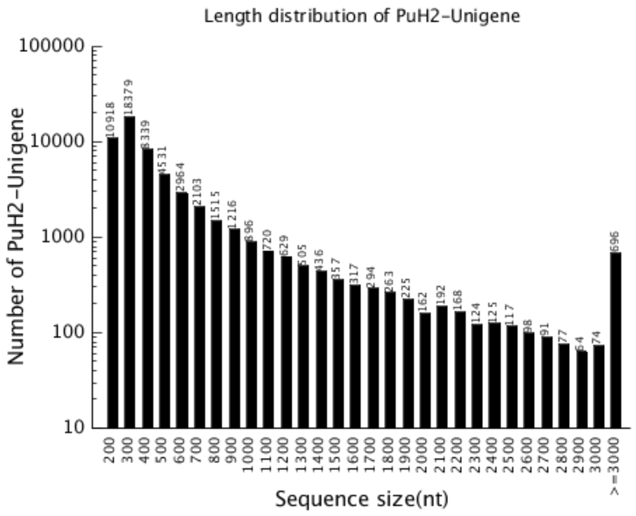
Length distribution of PuH2-Unigene
Number of PuH2-Unigene
Sequence size(nt)
10918 18379 8339 4531 2964 2103 1515 1216 896 720 629 505 436 357 317 294 263 225 162 192 168 124 125 117 98 91 77 64 74 696

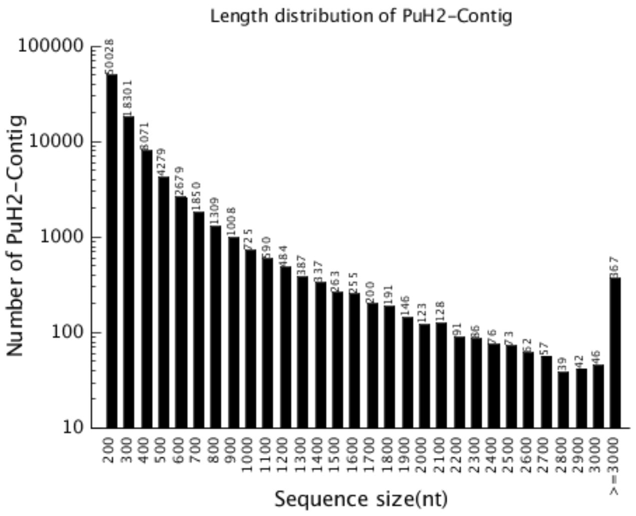
Length distribution of PuH2-Contig
Number of PuH2-Contig
Sequence size(nt)
50028 18301 8071 4279 2679 1850 1309 1008 725 590 484 387 337 263 255 200 191 146 123 128 91 86 76 73 62 57 39 42 46 367

Figure S2: Relative quantification of key genes using qPCR (columns) and RPKM values (line), throughout metamorphosis:

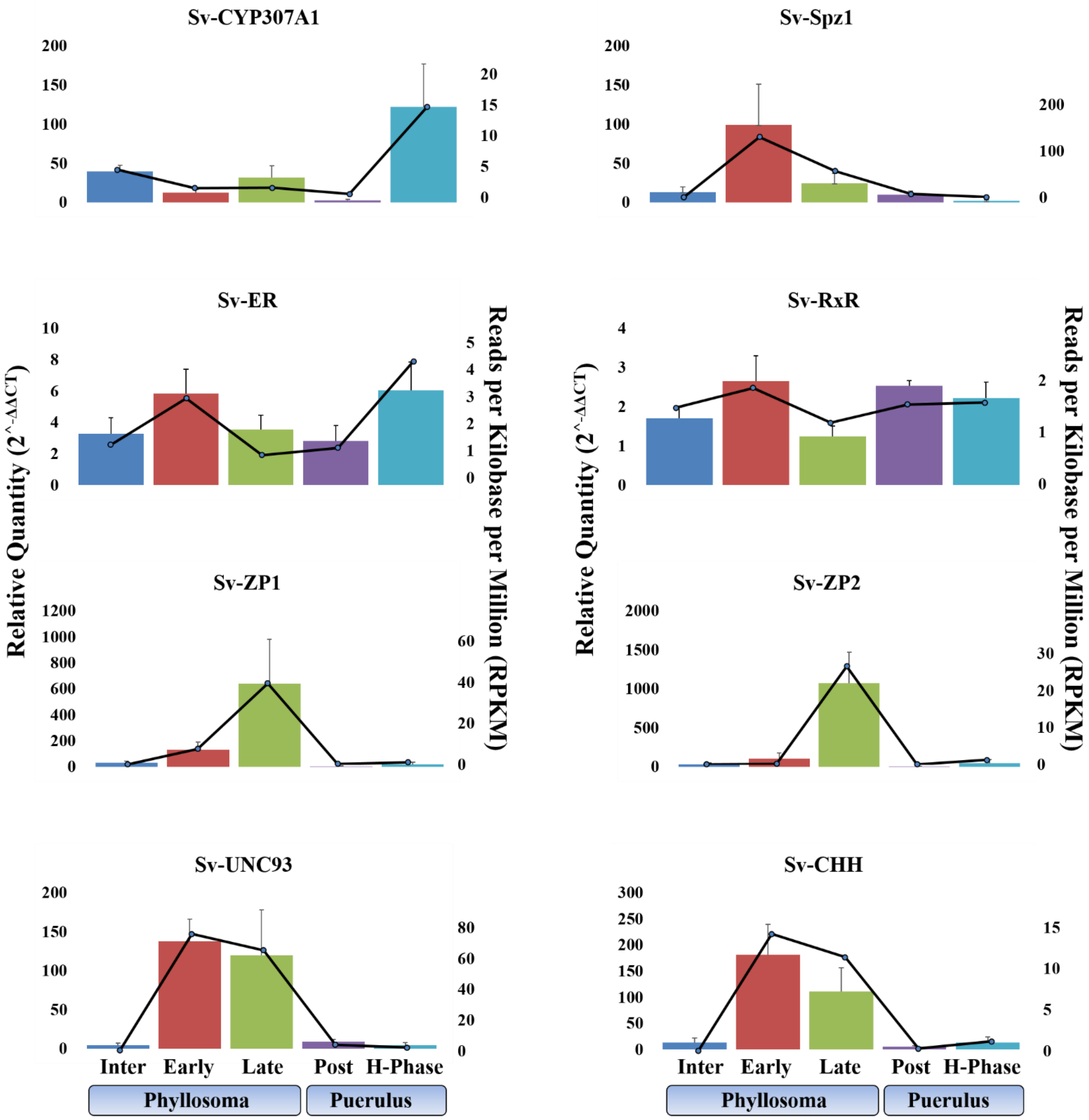

Details of genes, primers and probes used for qPCR analysis in Figure S2:

Gene name	GenBank accession no. / unigene	Forward primer (5' to 3')	Reverse primer (5' to 3')	Probe
Sv-18S	KF828103	ggtgcatggccgttctta	tggagatccgtcgactagttaat	22
Sv-FAMeT1	Unigene44448	ccacgttgccctcacttc	ttcttgaacctgatggctga	22
Sv-CYP15A1	CL6907.Contig1	cgtgggtcatggacgact	ttcagagtggcatccgtgta	1
Sv-CYP307A1	Unigene44916	ggaggatctcttgggtggat	agggtgttggaggatgtagc	156
Sv-ER	CL5835.contig4	aacagccagtaacatcccaaa	tcacactcaagaacaagcgact	142
Sv-RxR	CL4526.contig2	cagtcccttgtctccaccac	acagacatgacgggcttctc	157
Sv-ZP1	Unigene50269	agcgaccccaactgtgtg	agctgttgaggtagatgtcgaa	116
Sv-ZP2	Unigene30964	acccggcccagtcatagt	tggtggtgctgactggatta	78
Sv-UNC93	CL5857.Contig2	ccaggttggtgatgttggt	ctctcctgcggtgtcaactt	78
Sv-Spz1	CL5710.Contig1_All	ctaccgacatcccatgtaacac	agcagttctccagtcatctcg	86
Sv-CHH	Unigene30324	gagggaagccacgagagac	ggtgaacctgacaacaccaa	51
